# Significant predictors of clinical outcomes in metabolic associated fatty liver disease-related hepatocellular carcinoma following hepatectomy

**DOI:** 10.1097/MD.0000000000043248

**Published:** 2025-07-11

**Authors:** Pojen Hsiao, Kuang-Chun Hu, Wen-Lung Wang, Pei-Min Hsieh, Szu-Ying Chen, Steven Yu Lin, Hung-Yu Lin, Chen-Ti Wang, Yaw-Sen Chen, Yu-Wei Huang, Chih-Wen Lin

**Affiliations:** a Division of Gastroenterology and Hepatology, E-Da Hospital, I-Shou University, Kaohsiung, Taiwan; b Division of Gastroenterology and Hepatology, E-Da Dachang Hospital, I-Shou University, Kaohsiung, Taiwan; c Healthy Evaluation Center and Division of Gastroenterology, Department of Internal Medicine, MacKay Memorial Hospital, Taipei, Taiwan; d Mackay Junior College of Medicine, Nursing and Management, New Taipei, Taiwan; e School of Medicine, College of Medicine, I-Shou University, Kaohsiung, Taiwan; f Department of Surgery, E-Da Hospital, I-Shou University, Kaohsiung, Taiwan; g Department of Surgery, E-Da Dachang Hospital, I-Shou University, Kaohsiung, Taiwan; h Division of Occupational Medicine, E-Da Hospital, I-Shou University, Kaohsiung, Taiwan; i Division of Surgical Intensive Care, Department of Critical Care Medicine, E-Da Hospital, I-Shou University, Kaohsiung, Taiwan; j Department of Nursing, Fooyin University, Kaohsiung, Taiwan; k New York University, College of Arts and Science, New York, NY; l Department of Surgery, E-Da Cancer Hospital, I-Shou University, Kaohsiung, Taiwan; m Division of Endocrinology and Metabolism Department, E-Da Hospital, I-Shou University, Kaohsiung, Taiwan; n Department of Anesthesiology, Emergency and Critical Care Center, E-Da Hospital, I-Shou University, Kaohsiung, Taiwan; o School of Chinese Medicine, College of Chinese Medicine, and Research Center for Traditional Chinese Medicine, China Medical University, Taichung, Taiwan; p School Lin Da Clinic, Lin Da Health Group, Kaohsiung, Taiwan.

**Keywords:** ALDH2 rs671 polymorphism, hepatectomy, hepatocellular carcinoma, mortality, predictor, recurrence

## Abstract

The risk factors for clinical outcomes of hepatocellular carcinoma (HCC) associated with metabolic-associated fatty liver disease (MAFLD) following hepatectomy remain uncertain. The association between aldehyde dehydrogenase 2 (ALDH2) rs671 polymorphism and HCC recurrence and mortality remains unknown in this population. This retrospective cohort study enrolled 70 patients with MAFLD-related HCC who underwent hepatectomy between 2011 and 2022 at the E-Da Hospital, I-Shou University, Kaohsiung, Taiwan. Data analysis was completed in October 2023. The proposed criteria for the diagnosis of MAFLD are based on the evidence of hepatic steatosis (>5%) and one of the following 3 criteria: overweight/obesity, presence of type 2 diabetes mellitus, or evidence of metabolic dysregulation. The end points were HCC recurrence and overall mortality. Of the 70 patients enrolled, 55 (78.6%) were men, and the mean (standard deviation) age was 67.4 (8.0) years. HCC recurrence occurred in 19 patients, and 20 died. The 10-year cumulative HCC recurrence and mortality rates after resection were 55.4% and 43.9%, respectively. In Cox proportional analyses, patients with ALDH2 rs671 genotypes GA/AA had the highest HCC recurrence rate (hazard ratio [HR]: 3.48, 95% confidence interval [CI]: 1.08–12.4, *P* = .045), followed by those with multiple tumors (HR: 3.06, 95% CI: 1.11–10.9, *P* = .039). Moreover, patients with Barcelona clinic liver cancer stage B/C had the highest mortality rate (HR: 3.94, 95% CI: 1.34–11.6, *P* = .012), followed by those with hypertension (HR, 3.47; 95% CI: 1.28–9.40, *P* = .015). ALDH2 rs671 polymorphism and multiple liver tumors were strongly associated with HCC recurrence. Furthermore, Barcelona clinic liver cancer stage and hypertension were significantly associated with mortality in MAFLD-related HCC patients after hepatectomy.

## 1. Introduction

Hepatocellular carcinoma (HCC) is the second most common cause of cancer-related death worldwide.^[1–3]^ In Taiwan, HCC is often caused by viral and alcohol related diseases.^[4,5]^ However, it is difficult to diagnose early stage HCC, leading to high recurrence and mortality rates after surgery.^[6,7]^ Several prognostic factors for recurrence after HCC resection include tumor number and size, microvascular invasion, metabolic syndrome, cirrhosis, and Barcelona Clinic Liver Cancer (BCLC).^[6,8,9]^ However, the current tumor biomarkers used to predict prognosis after surgery for HCC are not very effective. Therefore, it is important to identify the predictors of HCC recurrence and mortality to improve the outcomes of patients undergoing surgery.

The diagnosis of metabolic-associated fatty liver disease (MAFLD) is based on the presence of liver steatosis, overweight/obesity, type 2 diabetes mellitus, or metabolic dysregulation. Metabolic dysregulation is characterized by at least two of the following risk factors: increased waist circumference, prediabetes, hypertension, hypertriglyceridemia, and low serum high-density lipoprotein cholesterol levels.^[10,11]^ Additionally, the prevalence of both cirrhosis and HCC in relation to MAFLD is on the rise.^[9,12–16]^ Several studies have compared the clinical prognoses of patients with MAFLD-, viral-, and alcohol-related HCC following surgical resection.^[9,12–14,17–19]^ However, there is limited information regarding the predictive factors for HCC recurrence and mortality in MAFLD-related HCC after hepatectomy.

One potential predictor is aldehyde dehydrogenase 2 (ALDH2) polymorphism, which affects the development of HCC in alcoholic liver disease.^[4,20,21]^ Our previous study found that heavy alcohol consumption and ALDH2 rs671 polymorphism significantly increased the risk of developing HCC and overall mortality in patients with hepatitis B virus-related cirrhosis.^[4]^ Moreover, another study indicated that ALDH2 rs671 genotype GG was associated with shorter overall survival but not recurrence-free survival in patients with viral-related HCC who underwent surgery.^[22]^ Furthermore, previous studies have shown that ALDH polymorphisms impair alcohol metabolism in patients with MAFLD, potentially leading to its progression.^[23–25]^ Specifically, ALDH2 polymorphisms have been linked to an increased probability of MAFLD and worsening of the condition.^[26,27]^ However, the impact of ALDH2 rs671 genotype on HCC recurrence and overall mortality in patients with MAFLD-related HCC after surgery remains uncertain and largely unknown. Therefore, this study aimed to identify the risk factors for HCC recurrence and overall mortality in MAFLD-related HCC patients who underwent surgery. We also examined the association between the ALDH2 rs671 polymorphism and HCC recurrence and mortality in this population.

## 2. Methods

### 2.1. Patients

This retrospective study enrolled 70 patients with MAFLD-related HCC who underwent hepatectomy at the E-Da Hospital, I-Shou University, Kaohsiung, Taiwan, between October 2011 and December 2022. These patients were regularly monitored through liver function tests, serum α-fetoprotein levels, and imaging examinations such as abdominal ultrasound, magnetic resonance imaging, and computed tomography. Clinical investigations were conducted every 3 to 6 months to detect HCC. The data analysis was completed on October 31, 2023.

### 2.2. Clinical procedures and evaluation criteria

The proposed criteria for diagnosing MAFLD were based on evidence of hepatic steatosis (>5%), along with one of the following 3 criteria: overweight/obesity (body mass index ≥ 23 kg/m² in Asians), the presence of type 2 diabetes mellitus, or evidence of metabolic dysregulation. Metabolic dysregulation was defined as having at least 2 of the following abnormalities: waist circumference ≥ 90 cm for men or ≥ 80 cm for women, blood pressure ≥ 130/85 mm Hg or specific drug treatment, plasma triglycerides ≥ 150 mg/dL or specific drug treatment, plasma high-density lipoprotein < 40 mg/dL for men or < 50 mg/dL for women or specific drug treatment, prediabetes, a homeostasis model assessment of insulin resistance score ≥ 2.5, and a plasma high-sensitivity C-reactive protein level > 2 mg/L.

However, this study excluded patients who had hepatitis B virus or hepatitis C virus infections, consumed > 20 g of alcohol per day, or experienced HCC recurrence after surgery. The presence of ALDH2 rs671 polymorphism was determined by blood analysis. ALDH2 polymorphism results in 3 different genotypes: GG, AA, and GA. Patients with GA and AA genotypes were grouped together as the GA/AA group to assess ALDH2 deficiency.

The primary endpoint of the study was HCC recurrence and the secondary endpoint was overall mortality. The follow-up time was defined as the duration from the data of inclusion in the study to mortality, last follow-up, or completion of the study (October 31, 2023), whichever occurred first. Recurrence time was defined as the duration from the date of inclusion to the date of HCC recurrence, mortality, last follow-up, or completion of the study, whichever occurred first. HCC recurrence was determined based on pathology or typical HCC imaging according to the guidelines of the American Association for the Study of Liver Disease.^[3]^ The study recorded various demographic and tumor characteristics, including clinical and pathological data. The study protocol was approved by the Institutional Review Board of E-Da Hospital (EMRP31112N). All the participants provided informed consent.

### 2.3. Statistical analysis

Continuous variables were expressed as mean and standard deviation and compared using the Student *t* test. Categorical variables are presented as numbers (percentages) and compared using the chi-squared test. Kaplan–Meier methodology and log-rank test were used to estimate the cumulative rates of HCC recurrence and overall mortality. Competing risk analyses were conducted to account for the fact that patients who died were no longer at a risk of HCC recurrence, estimating the risk of HCC recurrence while considering mortality as a competing risk. Both univariate and multivariate analyses were performed using Cox proportional regression models to identify the predictors associated with recurrent HCC and mortality. Statistical significance was defined as *P* < .05. All analyses were conducted using the Statistical Package for Social Sciences (SPSS, version 23.0; Chicago).

## 3. Results

### 3.1. Baseline demographic characteristics

Table 1 summarizes the demographic characteristics. Of the total participants, 55 (78.6%) were male, with an average age of 67.4 (standard deviation 8.0) years. Nineteen participants experienced HCC recurrence and 20 died during the study period. Additionally, 11.4% of the patients had liver cirrhosis. The majority of patients were classified as TNM stage I–II (88.6%) and BCLC stage 0–A (85.3%).

**Table 1:** Basic demographic data of all patients and correlations between ALDH2 polymorphisms and clinicopathologic features.

Characteristics	All patients (n = 70)	ALDH2 rs671 genotype		*P*-value
		GG (n = 26)	GA/AA (n = 44)	
Gender				
Female	15 (21.4)	7 (26.9)	8 (18.2)	.389
Male	55 (78.6)	19 (73.1)	36 (81.8)	
Age (years)	68 ± 8.0	68.9 ± 8.1	66.5 ± 7.9	.218
BMI (kg/m^2^)	26.2 ± 3.4	26.5 ± 3.5	26.1 ± 3.4	.662
HTN	35 (50.0)	15 (57.7)	20 (45.5)	.322
DM	26 (37.1)	11 (42.3)	15 (34.1)	.492
Hyperlipidemia	47 (67.1)	16 (61.5)	31 (70.5)	.443
Smoking	7 (10.0)	1 (3.8)	6 (13.6)	.187
AST (IU/L)	46 ± 32	39 ± 16	51 ± 38	.135
ALT (IU/L)	44 ± 39	35 ± 20	49 ± 47	.151
Total bilirubin (mg/dL)	0.9 ± 0.5	0.8 ± 0.3	0.9 ± 0.6	.171
Albumin (g/dL)	4.2 ± 0.3	4.1 ± 0.3	4.3 ± 0.3	.029
Creatinine	1.3 ± 0.8	1.4 ± 0.3	1.3 ± 0.3	.330
Platelet count (x10^3^/mL)	195 ± 64	183 ± 65	202 ± 65	.217
INR	1.0 ± 0.1	1.0 ± 0.1	1.0 ± 0.1	.818
AFP (ng/dL)	2918 ± 15461	822 ± 3074	4157 ± 19335	.387
ICG (%)	11.8 ± 6.6	12.8 ± 6.6	11.3 ± 6.6	.356
Liver cirrhosis				
Absent	62 (88.6)	24 (92.3)	38 (86.4)	.450
Present	8 (11.4)	2 (7.7)	6 (13.6)	
Child-Pugh class				
Class A	69 (98.6)	26 (100)	43 (97.7)	.439
Class B	1 (1.4)	0 (0)	1 (2.3)	
Operative margin (>1 cm)				
Absent	48 (68.6)	19 (73.1)	29 (65.9)	.533
Present	22 (31.4)	7 (26.9)	15 (34.1)	
Edmondson-Steiner Grades				
I–II	66 (94.3)	25 (96.2)	41 (93.2)	.794
III–IV	4 (5.7)	1 (3.8)	3 (6.8)	
Macrovascular invasion				
Absent	64 (91.4)	22 (84.6)	42 (95.5)	.118
Present	6 (8.6)	14 (12.6)	2 (4.5)	
Microvascular invasion				
Absent	57 (81.4)	21 (80.8)	36 (81.8)	.913
Present	13 (18.6)	5 (19.2)	8 (18.2)	
Lymph node invasion				
Absent	69 (98.6)	26 (100)	43 (97.7)	.439
Present	1 (1.4)	0 (0)	1 (2.3)	
Tumor number				
Single	66 (94.3)	25 (96.2)	41 (93.2)	.605
Multiple	4 (5.7)	1 (3.8)	3 (6.8)	
Tumor size (cm)	5.2 ± 2.8	5.1 ± 2.8	5.3 ± 2.8	.759
Tumor size				
< 5 cm	39 (55.7)	15 (57.5)	24 (54.5)	.798
≥ 5 cm	31 (44.3)	11 (42.3)	20 (45.5)	
TNM stage				
I–II	62 (88.6)	22 (85.6)	40 (80.9)	.534
III–IV	8 (11.4)	4 (15.4)	4 (9.1)	
BCLC stage				
0–A	61 (85.3)	23 (84.6)	39 (88.6)	.397
B–C	9 (14.7)	4 (15.4)	5 (11.4)	
Recurrence				
Absent	51 (72.9)	23 (88.5)	28 (63.6)	.024
Present	19 (27.1)	3 (11.5)	16 (36.4)	
Recurrence time	4.0 ± 2.9	4.4 ± 3.2	3.8 ± 2.5	.022
Mortality				
	50 (71.4)	19 (73.1)	31 (70.5)	.814
Present	20 (29.5)	7 (26.9)	13 (29.5)	
Follow up time	5.1 ± 3.1	4.8 ± 3.5	5.2 ± 2.9	.631

Data shown as mean ± standard deviation or number (%).

AFP = alpha-fetoprotein, ALDH2 = aldehyde dehydrogenase 2, ALT = alanine aminotransferase, AST = aspartate aminotransferase, BCLC stage = Barcelona clinic liver cancer, BMI = body mass index, DM = diabetes mellitus, HTN = hypertension, ICG = indocyanine green, INR = international normalized ratio.

### 3.2. ALDH2 rs671 polymorphism is correlated with tumor recurrence

Among the 70 patients, 37.1% (26) had the ALDH2 rs671 GG genotype, while 62.9% (44) had the GA/AA genotype (Table 1). The ALDH2 rs671 genotype was significantly correlated with tumor recurrence (3 [11.5%] vs 16 [36.3%], *P* = .024) and serum albumin levels (4.1 ± 0.3, 4.3 ± 0.3, *P* = .029).

### 3.3. Predictive factors correlated with tumor recurrence in HCC patients underwent surgical resection

**Figure 1.**
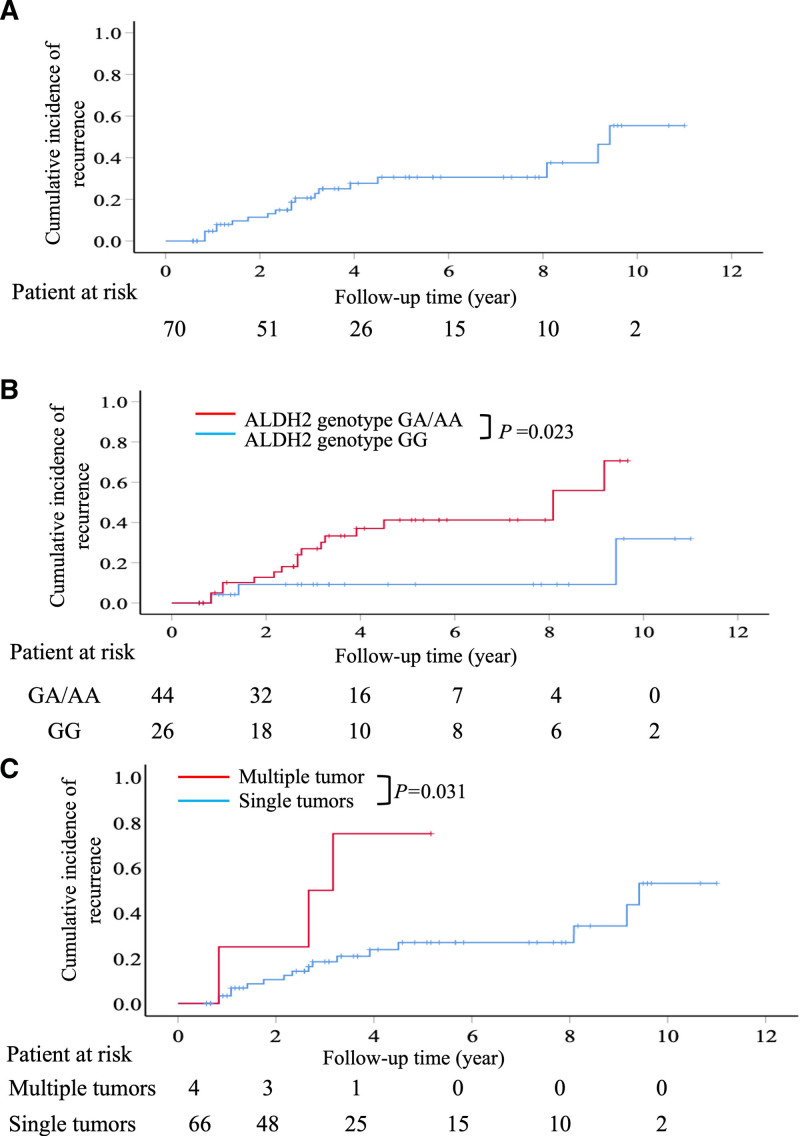
The cumulative incidences of hepatocellular carcinoma recurrence after surgical resection. (A) Cumulative incidence of HCC recurrence in all patients. (B) Cumulative incidence of HCC recurrence according to the ALDH2 rs671 polymorphism. Patients with GA/AA genotypes were significantly correlated with an increased incidence of HCC recurrence compared to those with the GG genotype. (C) Cumulative incidence of HCC recurrence according to tumor number. The incidence of HCC recurrence was significantly higher in patients with multiple liver tumors than in those with a single liver tumor. ALDH2 = aldehyde dehydrogenase 2, HCC = hepatocellular carcinoma.

In this study, HCC recurrence was observed in 19 patients who had undergone hepatectomy. The cumulative incidences of HCC recurrence at 1, 3, 5, and 10 years were 4.7%, 20.6%, 30.6%, and 55.4%, respectively (Fig. 1A). Univariate analysis revealed that ALDH2 rs671 genotype GA/AA and multiple liver tumors were significantly associated with a higher rate of HCC recurrence (Table 2). Additionally, multivariate Cox regression analysis demonstrated that patients with the ALDH2 rs671 genotype GA/AA had the highest HCC recurrence rate (hazard ratio [HR]: 3.48, 95% confidence interval [CI]: 1.08–12.4, *P* = .039), followed by those with multiple liver tumors (HR: 3.06, 95% CI: 1.11–10.9, *P* = .045) (Table 2).

**Table 2:** Univariate and multivariate analyses of factors associated with tumor recurrence of hepatocellular carcinoma patients who underwent curative resection.

Characteristics	Univariate analyses		Multivariate analyses	
	HR (95% CI)	*P*-value	HR (95% CI)	*P*-value
Gender, Female vs Male	2.19 (0.63–7.62)	.218		
Age (years), <65 vs ≥65	0.48 (0.19–1.22)	.122		
Body Mass Index, (kg/m^2^)	0.97 (0.35–2.69)	.946		
Diabetes Mellitus, Absent vs Presence	0.90 (0.51–1.6)	.715		
Hypertension, Absent vs Presence	0.98 (0.39–2.43)	.968		
Hyperlipidemia, Absent vs Presence	1.16 (0.42–3.24)	.771		
Smoking, Absent vs Presence	1.76 (0.39–7.83)	.455		
AST (IU/L), <40 vs ≥40	2.47 (0.96–6.32)	.060		
ALT (IU/L), <40 vs ≥40	1.38 (0.55–3.43)	.490		
Total Bilirubin (mg/dL), <1.2 vs ≥1.2	1.12 (0.32–3.85)	.866		
Albumin (g/dL), <3.5 vs ≥3.5	0.28 (0.02–1.47)	.111		
Platelet count (x10^3^/mL), <100K vs ≥100K	1.37 (0.39–4.8)	.626		
INR, <1.0 vs ≥1.0	1.39 (0.53–3.71)	.500		
AFP (ng/dL) < 200 vs ≥200	1.19 (0.35–4.12)	.777		
Liver cirrhosis, Absent vs Presence	1.79 (0.59–5.39)	.305		
Child-Pugh class, A vs B	2.08 (0.28–3.58)	.720		
Operative margin (cm), <1.0 vs ≥1.0	1.53 (0.59–3.94)	.377		
Tumor number, Single vs Multiple	4.07 (1.15–14.3)	.029	3.06 (1.11–10.9)	.039
Tumor size (cm), <5 vs ≥5	1.18 (0.47–2.97)	.719		
Edmondson-Steiner Grade I–II vs III–IV	1.69 (0.51–4.98)	.412		
Macrovascular invasion, Absent vs Presence	25.0 (0.06–125)	.280		
Microvascular invasion, Absent vs Presence	1.88 (0.43–8.33)	.402		
Lymph node invasion, Absent vs Presence	20.0 (0.67–166)	.849		
TNM stage, I–II vs III–IV	1.22 (0.28–5.34)	.794		
BCLC stage, 0–A vs B–C	1.88 (0.53–6.68)	.327		
ALDH2 rs671 genotype, GG vs GA/AA	3.85 (1.10–13.4)	.035	3.48 (1.08–12.4)	.045

AFP = alpha-fetoprotein, ALDH2 = aldehyde dehydrogenase 2, ALT = alanine aminotransferase, AST = aspartate aminotransferase, BCLC stage = Barcelona clinic liver cancer, CI = confidence interval, HR = hazard ratio, INR = International normalized ratio.

Kaplan–Meier analysis revealed that patients with the ALDH2 rs671 genotype GA/AA had a significantly higher rate of recurrent HCC than those with the ALDH2 rs671 genotype GG. Patients with the GA/AA genotype had cumulative HCC recurrence rates of 5%, 27%, 41.2%, and 70.6% at 1, 3, 5, and 10 years, respectively, whereas those with the GG genotype had recurrence rates of 4.2%, 9.2%, 9.2%, and 31.9%, respectively (Fig. 1B).

Furthermore, Kaplan–Meier analysis showed that patients with multiple liver tumors had a significantly higher rate of recurrent HCC than those with a single liver tumor. Patients with multiple liver tumors had cumulative HCC recurrence rates of 25%, 50%, 75%, and 75% at 1, 3, 5, and 10 years, respectively, whereas patients with a single liver tumor had recurrence rates of 6.8%, 20.8%, 26.9%, and 53%, respectively (Fig. 1C).

**Figure 2.**
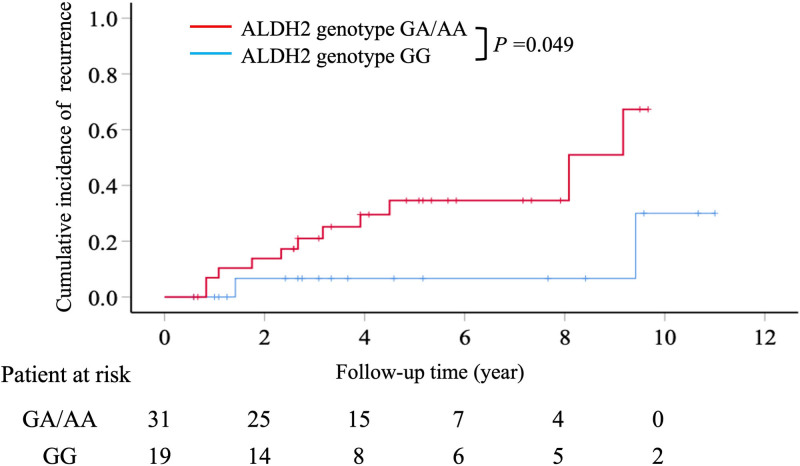
The cumulative incidences of hepatocellular carcinoma recurrence after surgical resection after competing risk analysis. After competing risk analysis, patients with GA/AA genotypes were notably associated with an increased incidence of HCC recurrence compared with those with the GG genotype. HCC = hepatocellular carcinoma.

The rate of recurrent HCC was significantly higher in patients with the ALDH2 rs671 GA/AA genotype than in those with the GG genotype (*P* = .049; Fig. 2) in the competing risk analysis. Patients with the GA/AA genotype had cumulative HCC recurrence rates of 6.9%, 21%, 34.6%, and 67.3% at 1, 3, 5, and 10 years, respectively, whereas those with the GG genotype had recurrence rates of 0%, 6.7%, 6.7%, and 30%, respectively (Fig. 2).

### 3.4. Predictive factors correlated with mortality in HCC patients underwent surgical resection

**Figure 3.**
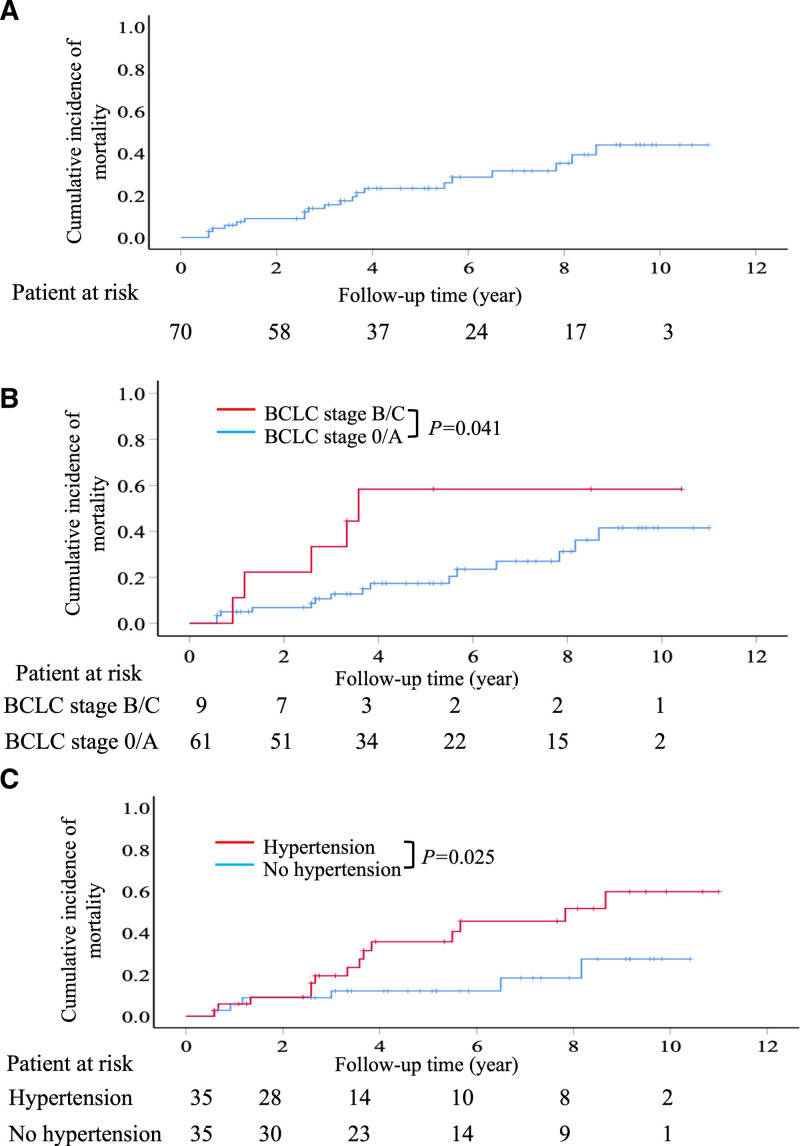
The cumulative incidences of mortality after surgical resection. (A) Cumulative incidence of mortality in all patients. (B) Cumulative incidence of mortality according to the BCLC stage. Patients with BCLC stages B/C had a significantly increased incidence of mortality compared to those with BCLC stage 0/A. (C) Patients with hypertension had a significantly higher incidence of mortality than those without hypertension. BCLC = Barcelona clinic liver cancer.

During the average follow-up period of 5.1 years, 20 patients died. The cumulative mortality rates 1, 3, 5, and 10 years after hepatectomy were 5.8%, 13.8%, 23.3%, and 43.9%, respectively (Fig. 3A). Univariate analysis revealed that several factors were significantly associated with increased mortality, including hypertension and BCLC stage B–C (Table 3). Multivariate Cox regression analysis demonstrated that patients with BCLC stage B/C had the highest mortality rate (HR: 3.94, 95% CI: 1.34–11.6, *P* = .012), followed by those with hypertension (HR: 3.47, 95% CI: 1.28–9.40, *P* = .015) (Table 3).

**Table 3:** Univariate and multivariate analyses of factors associated with mortality of hepatocellular carcinoma patients who underwent curative resection.

Characteristics	Univariate analyses		Multivariate analyses	
	HR (95% CI)	*P*-value	HR (95% CI)	*P*-value
Gender, Female vs Male	2.15 (0.63–7.38)	.223		
Age (years), <65 vs ≥65	1.10 (0.45–2.69)	.834		
Body Mass Index, (kg/m^2^)	0.55 (0.22–1.36)	.197		
Diabetes Mellitus, Absent vs Presence	1.10 (0.45–2.68)	.832		
Hypertension, Absent vs Presence	2.76 (1.06–7.18)	.038	3.47 (1.28–9.40)	.015
Hyperlipidemia, Absent vs Presence	0.57 (0.23–1.41)	.226		
Smoking, Absent vs Presence	2.23 (0.74–6.69)	.153		
AST (IU/L), <40 vs ≥40	1.96 (0.78–4.93)	.155		
ALT (IU/L), <40 vs ≥40	1.48 (0.61–3.58)	.383		
Total Bilirubin (mg/dL), <1.2 vs ≥1.2	2.28 (0.86–6.05)	.098		
Albumin (g/dL), <3.5 vs ≥3.5	0.67 (0.22–2.00)	.469		
Platelet count (x10^3^/mL), <100K vs ≥100K	0.13 (0.02–1.06)	.057		
Prothrombin time, <1.0 vs ≥1.0	2.63 (0.76–9.08)	.126		
AFP (ng/dL) < 200 vs ≥200	1.06 (0.31–3.57)	.926		
Liver cirrhosis, Absent vs Presence	1.96 (0.65–5.87)	.230		
Child-Pugh class, A vs B	5.05 (0.65–39.2)	.121		
Operative margin (cm), <1.0 vs ≥1.0	1.07 (0.42–2.65)	.908		
Tumor number, Single vs Multiple	2.07 (0.48–8.96)	.333		
Tumor size (cm), <5 vs ≥5	2.22 (0.91–5.44)	.082		
Edmondson-Steiner Grade I–II vs III–IV	2.35 (0.78–7.11)	.130		
Macrovascular invasion, Absent vs Presence	1.78 (0.56–5.13)	.389		
Microvascular invasion, Absent vs Presence	1.44 (0.47–4.38)	.520		
Lymph node invasion, Absent vs Presence	16.3 (0.96–145)	.056		
TNM stage, I–II vs III–IV	2.76 (0.99–7.64)	.051		
BCLC stage, 0–A vs B–C	3.05 (1.10–8.42)	.032	3.94 (1.34–11.6)	.012
Recurrence, Absent vs Presence	1.52 (0.57–3.98)	.398		
ALDH2 rs671 genotype, GG vs GA/AA	1.18 (0.63–2.19)	.607		

AFP = alpha-fetoprotein, ALDH2 = aldehyde dehydrogenase 2, ALT = alanine aminotransferase, AST = aspartate aminotransferase, BCLC stage = Barcelona clinic liver cancer, CI = confidence interval, HR = hazard ratio, INR = International normalized ratio.

Patients with BCLC stages B–C had a significantly higher mortality rate than those with BCLC stage 0/A. Among patients with BCLC stage B–C, the mortality rates at 1, 3, 5, and 10 years were 5.0%, 10.6%, 17.3%, and 41.5%, respectively. In contrast, patients with BCLC stage 0/1 had mortality rates of 1.1%, 33.3%, 58.3%, and 58.3% at 1, 3, 5, and 10 years, respectively (Fig. 3B).

Additionally, patients with hypertension have a significantly higher mortality rate than those without. Among the patients with hypertension, the mortality rates at 1, 3, 5, and 10 years were 5.9%, 19.4%, 35.8%, and 59.7%, respectively. In contrast, those without hypertension had mortality rates of 5.8%, 12.1%, 12.1%, and 27.4% at 1, 3, 5, and 10 years, respectively (Fig. 3C).

## 4. Discussion

This study aimed to identify risk factors for recurrent HCC and overall mortality in patients with MAFLD-related HCC who underwent hepatectomy. This study included 70 patients and found that ALDH2 rs671 genotype GA/AA and multiple liver tumors were associated with higher rates of HCC recurrence. Furthermore, this study identified BCLC stages B/C and hypertension as significant risk factors for mortality. These findings suggest that the ALDH2 rs671 polymorphism could be a valuable predictor of recurrent HCC but not mortality in patients with MAFLD-related HCC after resection. This study is the first to highlight a significant correlation between the ALDH2 rs671 polymorphism and recurrent HCC in patients with MAFLD-related HCC after hepatectomy.

Our previous study demonstrated that the ALDH2 rs671 genotype, combined with heavy alcohol consumption, significantly influenced the development of HCC and mortality in patients with alcohol-related cirrhosis.^[4]^ Interestingly, the ALDH2 rs671 GG genotype effectively metabolizes alcohol, thereby reducing the likelihood of carcinogenic acetaldehyde accumulation.^[4,22]^ Furthermore, previous studies have shown that ALDH polymorphisms impair alcohol metabolism in patients with MAFLD, potentially leading to its progression.^[23–25]^ Specifically, ALDH2 polymorphisms increase the probability of developing MAFLD and exacerbate the condition of MAFLD.^[26,27]^ The current study also revealed that the ALDH2 rs671 GA/AA genotype was associated with an increased risk of HCC recurrence but not mortality in patients with MAFLD-related HCC after resection. Therefore, the ALDH2 rs671 polymorphism not only increases the risk of HCC recurrence in alcoholic liver disease-related HCC patients after resection but also poses a risk for HCC recurrence in patients with MAFLD-related HCC after resection. Further studies are required to validate our results.

Risk factors for clinical outcomes in patients with viral-related HCC after hepatectomy have been extensively studied.^[9,12,14]^ However, limited information is available regarding the risk factors for clinical prognosis in patients with MAFLD-related HCC after hepatectomy.^[18]^ Our study revealed that patients with multiple liver tumors had a significantly higher rate of recurrent HCC than those with a single liver tumor after hepatectomy for MAFLD-related HCC. Multiple liver tumors have been identified as high-risk factors for HCC recurrence after hepatectomy. This result differs slightly from those of previous studies showing that male sex, satellite nodules, tumor size, and microvascular invasion are significantly associated with tumor recurrence in patients with MAFLD-related HCC.^[18]^ Furthermore, our study demonstrated that patients with BCLC stage B/C disease and hypertension had higher mortality rates than those with MAFLD-related HCC after hepatectomy. Patients with BCLC stages B/C had a higher mortality rate than those with BCLC stage 0/A, indicating that tumor stage and liver function preservation are associated with poor prognosis in patients with MAFLD-related HCC after hepatectomy. These results are consistent with previous studies showing that cirrhosis, multiple tumors, and tumor size appear to be independent predictors of overall survival in patients with MAFLD-related HCC.^[13]^ Additionally, patients with hypertension had a higher mortality rate than those without hypertension among MAFLD-related HCC patients after hepatectomy, suggesting that comorbidity with hypertension is associated with poor prognosis.

In summary, ALDH2 rs671 polymorphism and multiple liver tumors were strongly associated with increased HCC recurrence in patients with MAFLD-related HCC following hepatectomy. Furthermore, patients with BCLC stages B/C and hypertension exhibited a higher mortality rate in this population. This study is the first to establish a clear link between the ALDH2 rs671 polymorphism and HCC recurrence in MAFLD-related HCC after hepatectomy. Therefore, clinicians should evaluate ALDH2 rs671 polymorphism to assess the risk of HCC recurrence in patients with MAFLD-related HCC following surgical resection.

## Author contributions

**Conceptualization:** Kuang-Chun Hu, Steven Yu Lin.

**Data curation:** Pojen Hsiao, Wen-Lung Wang, Yaw-Sen Chen.

**Formal analysis:** Pojen Hsiao, Pei-Min Hsieh, Chen-Ti Wang.

**Funding acquisition:** Pojen Hsiao, Yaw-Sen Chen, Chih-Wen Lin.

**Investigation:** Hung-Yu Lin, Chih-Wen Lin.

**Methodology:** Wen-Lung Wang, Szu-Ying Chen.

**Project administration:** Pei-Min Hsieh, Yu-Wei Huang.

**Resources:** Yaw-Sen Chen, Chih-Wen Lin.

**Software:** Steven Yu Lin, Hung-Yu Lin.

**Supervision:** Chih-Wen Lin.

**Validation:** Szu-Ying Chen, Yu-Wei Huang.

**Visualization:** Szu-Ying Chen, Yu-Wei Huang.

**Writing – original draft:** Pojen Hsiao, Kuang-Chun Hu, Hung-Yu Lin, Chen-Ti Wang, Chih-Wen Lin.

**Writing – review & editing:** Chen-Ti Wang, Yaw-Sen Chen, Yu-Wei Huang, Chih-Wen Lin.
